# Rapid genetic divergence in response to 15 years of simulated climate change

**DOI:** 10.1111/gcb.12966

**Published:** 2015-08-27

**Authors:** Catherine H. Ravenscroft, Raj Whitlock, Jason D. Fridley

**Affiliations:** ^1^ Department of BiologySyracuse University107 College PlaceSyracuseNY 13244USA; ^2^ Institute of Integrative BiologyUniversity of LiverpoolThe Biosciences Building, Crown StreetLiverpoolL69 7ZBUK; ^3^ Department of Animal and Plant SciencesUniversity of SheffieldWestern BankSheffieldS10 2TNUK

**Keywords:** adaptation, climate change, evolution, genetic differentiation, grassland

## Abstract

Genetic diversity may play an important role in allowing individual species to resist climate change, by permitting evolutionary responses. Our understanding of the potential for such responses to climate change remains limited, and very few experimental tests have been carried out within intact ecosystems. Here, we use amplified fragment length polymorphism (AFLP) data to assess genetic divergence and test for signatures of evolutionary change driven by long‐term simulated climate change applied to natural grassland at Buxton Climate Change Impacts Laboratory (BCCIL). Experimental climate treatments were applied to grassland plots for 15 years using a replicated and spatially blocked design and included warming, drought and precipitation treatments. We detected significant genetic differentiation between climate change treatments and control plots in two coexisting perennial plant study species (*Festuca ovina* and *Plantago lanceolata*). Outlier analyses revealed a consistent signature of selection associated with experimental climate treatments at individual AFLP loci in *P. lanceolata*, but not in *F. ovina*. Average background differentiation at putatively neutral AFLP loci was close to zero, and genomewide genetic structure was associated neither with species abundance changes (demography) nor with plant community‐level responses to long‐term climate treatments. Our results demonstrate genetic divergence in response to a suite of climatic environments in reproductively mature populations of two perennial plant species and are consistent with an evolutionary response to climatic selection in *P. lanceolata*. These genetic changes have occurred in parallel with impacts on plant community structure and may have contributed to the persistence of individual species through 15 years of simulated climate change at BCCIL.

## Introduction

Climate change is expected to impose strong directional selection pressures on plant populations (Davis & Woods, 1986; Davis & Shaw, 2001; Jump & Peñuelas, 2005; Bradshaw & Holzapfel, 2006; Reusch & Wood, 2007; Anderson *et al*., 2012). In response, plant populations may adapt *in situ* via selection on standing genetic variation (Hoffmann & Willi, 2008; Jump *et al*., 2008a). This adaptive response could be an important component of species' resistance to climate change, because it would provide an *in situ* ‘option’ for persistence in spite of environmental change (Jump *et al*., 2008a).

Plant populations are often adapted to their local environments (Leimu & Fischer, 2008). These evolutionary responses can occur at fine spatial scales, despite gene flow. For example, populations of the grass *Anthoxanthum odoratum* show adaptive responses to heavy metal contamination at the boundaries of metal ore mines (Antonovics & Bradshaw, 1970) and have evolved in response to sharp boundaries separating grassland management regimes in the Park Grass Experiment (Snaydon & Davies, 1976; Gould *et al*., 2014). Genetic differentiation in response to gradients of climate (e.g. temperature) and climate‐driven abiotic factors (e.g. water availability) has been observed in plants at both fine spatial scales (Kelly *et al*., 2003; Parisod & Christin, 2008; Jump *et al*., 2009; Manel *et al*., 2010; Franks, 2011) and over landscapes (Hamrick & Allard, 1972; Hamrick & Holden, 1979; Li *et al*., 1999; Owuor *et al*., 2003; Jump *et al*., 2006). In some cases, associations between the climate and genetic structure are repeated at different spatial scales or with temporal climate changes (e.g. Hamrick & Allard, 1972; Hamrick & Holden, 1979; Jump *et al*., 2006). These patterns of genetic structuring are highly indicative of adaptive differentiation in response to environmental selection. More recently, genetic responses to the climate have been identified using genomic and quantitative genetic approaches (e.g. Jump *et al*., 2008b; Franks, 2011). Together, these studies indicate that climatic factors can act as potent forces of selection, driving adaptive differentiation between populations, and within populations at fine spatial scales despite potentially high levels of gene flow.

Although previous studies suggest adaptive responses to environmental selection, there have been very few direct experimental tests of genetic responses to climate change in plant species within intact ecosystems (Jump *et al*., 2008b; Avolio *et al*., 2013). Such tests are important, because they tell us about the capacity for neutral or adaptive genetic change in ecologically realistic settings that may incorporate competition from coexisting species, or fine‐scale abiotic heterogeneity that interferes with climatic selection. Avolio *et al*. (2013) used AFLP markers to demonstrate changes in clonal structure of the grass *Andropogon gerardii* in response to manipulation of precipitation regimes in a prairie ecosystem, but did not identify a clear adaptive component to these changes. Jump *et al*. (2008b) used an outlier analysis to identify adaptive divergence in response to simulated drought and warming in *Fumana thymifolia* within a Mediterranean shrub community, focussing on the establishing (seedling) phase of the plant life cycle. Thus, we know very little regarding either the extent to which climatic selection can drive adaptive divergence within plant populations occupying intact ecosystems, or about adaptive responses in the established (reproductively mature) phase of perennial plant species.

**Figure 1:**
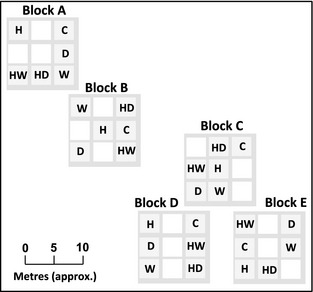
Layout of experimental plots at BCCIL. Each climate treatment is replicated five times in a randomized block design. C: Control, D: Drought, H: Heated, W: Watered, HD: Heated‐Drought, HW: Heated‐Watered.

At the Buxton Climate Change Impacts Laboratory (BCCIL) in northern England, intact species‐rich limestone grassland has been subjected to experimentally manipulated climate treatments since 1993 (involving increased temperature, modified precipitation and factorial combinations of these; Fig. 1). BCCIL maintains some of the longest‐running multifactorial climate manipulations in the world. Community composition has remained relatively stable in all experimental treatments (Grime *et al*., 2000, 2008). Such stability is rare; the majority of experimental climate manipulation studies report rapid community and ecosystem responses (e.g. Harte & Shaw, 1995; Grime *et al*., 2000; Zavaleta *et al*., 2003; Evans *et al*., 2011). Genetic adaptation to climatic selection has been hypothesized as one potential mechanism supporting the apparent resistance of this grassland community to long‐term climate manipulations (Grime *et al*., 2008). Genetic variation in phenotype has been documented at fine spatial scales (< 10 m) in several of the species that are common at BCCIL, and this diversity can influence plant community structure (Booth & Grime, 2003; Fridley *et al*., 2007; Whitlock *et al*., 2007, 2010; Bilton *et al*., 2010). Recently, Ravenscroft *et al*. (2014) have shown that subpopulations of *Plantago lanceolata* subjected to long‐term experimental climate treatments at BCCIL differed significantly in phenotype when grown under common environment conditions. This finding suggests that plant phenotypes have evolved in response to simulated climate change treatments and may be consistent with an adaptive response to the climatic environment.

We used amplified fragment length polymorphism (AFLP) markers to test for genetic differentiation in coexisting populations of established individuals of *P. lanceolata* and *Festuca ovina* exposed to 15 years of experimental climate manipulations at BCCIL. Simulated climate change could lead to genetic differentiation in this system through two processes: genetic drift and ecologically adaptive evolution. Genetic drift may be induced if a novel climate leads to altered demography (specifically, changes in subpopulation size or modified gene flow between treatments and individual plots; Ellstrand & Elam, 1993). The effects of drift are expected to be genomewide, leading to a consistent pattern of genetic differentiation across loci. Adaptive responses, on the other hand, are expected to accrue through selection on variation at individual gene loci that control adaptive phenotypes. In this case, we would expect to see a pattern of excess (outlier) differentiation at a limited number of selected loci against a background of genomewide differentiation imposed by drift. Crucially, neutral spatial genetic structure is not expected to lead to genetic structuring among climate treatments at BCCIL, as treatment plots are evenly replicated over spatial blocks in this experiment (Fig. 1). We combined multivariate and outlier analyses of genetic data with information on species‐level demography and abundance to assess first whether 15 years of simulated climate change had resulted in genetic differentiation and, second, whether genetic change was consistent with an evolutionary (adaptive) response to climatic selection.

## Materials and methods

### Study system and focal species

Prior to the onset of experimentation in 1993, the vegetation at the BCCIL site comprised an ancient, unimproved sheep pasture located on the side of a dale (valley) near Harpur Hill, Buxton, UK. The experiment now running at BCCIL was set up as a series of 3 × 3 m plots within this pasture, each containing intact species‐rich limestone grassland vegetation. These plots have been exposed to annual climatic treatments since 1993 using a replicated, spatially blocked design. Treatments include (i) winter warming, where soil surface is maintained at 3 °C above ambient (nonmanipulated) temperature from November to April; (ii) drought treatments, where rainfall in July and August is intercepted by automated rain shelters; (iii) water addition (watered), where water is added to experimental plots from June to September at a rate of 20% above the long‐term average; (iv) warming and drought (heated/drought); (v) warming and increased precipitation (heated/watered); and (vi) control. Experimental plots (3 × 3 m) are replicated five times in a randomized block design (Fig. 1). Grime *et al*. (2000) provide further details on the experimental design at BCCIL. Soil depth is a major source of fine‐scale environmental heterogeneity within plots, varying from 0 cm (bare rock) to > 30 cm, and influences plant community structure at local scales (Fridley *et al*., 2011).

**Figure 2:**
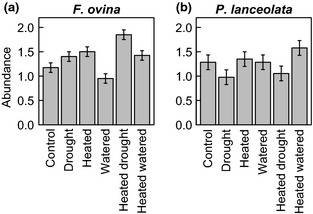
Species‐level abundance of *F. ovina* and *P. lanceolata* within simulated climate change treatments at BCCIL (data from Fridley *et al*., 2011). Abundance was measured as ordinal vegetation cover class (0 = 0–4%; 1 = 5–24%; 2 = 25–49%; 3 = 50–74%; 4 = 75% +) within 10 × 10 cm permanent quadrats located within each 3 × 3 m grassland plot (eight quadrats per plot, five plots per treatment). Error bars show one standard error of the mean.

*Festuca ovina* L. (sheep's fescue, a grass) and *Plantago lanceolata* L. (ribwort plantain, a forb) are perennial (polycarpic) plants with wide geographical distributions; both species are self‐incompatible (obligate outcrossers) and wind pollinated. Both study species are common in all experimental plots at BCCIL, and both have a limited capacity for clonal growth: *P. lanceolata* via lateral rosette formation and *F. ovina* through tillering, typically leading to the formation of loose tussocks. *Festuca ovina* was both the most frequent of all the species (occurring in 223 of 240 permanent 10 × 10 cm quadrats distributed throughout the grassland at BCCIL) and the species with greatest cover in the plots at BCCIL (Fridley *et al*., 2011). This species was significantly more abundant in heated, drought‐treated and heated/drought plots and less abundant in watered plots after 15 years of climatic manipulation (Fridley *et al*., 2011; Fig. 2a). Soil depth was not a significant predictor of *F. ovina* abundance (Fridley *et al*., 2011). The abundance of *P. lanceolata* varied with climate treatment, soil depth and a treatment × soil depth interaction (Fridley *et al*., 2011). The abundance of this species was highest in warming treatments, was lowest in treatments that included drought and varied considerably with soil depth in all experimental treatments (Fridley *et al*., 2011; Fig. 2b).

### Sample collection

In the following, we use ‘total population’ or ‘population’ to refer to the individuals of a species present across the BCCIL site. Subsets of individuals within climate treatments, blocks or plots are referred to as ‘subpopulations’. We collected leaf tissue samples of each species from control, drought‐treated, warmed, watered, heated‐drought and heated‐watered plots at BCCIL in June 2009 (30 plots in total), after 15 years of experimental treatments (further details are given in Text S2). We sampled 12 individuals of each species from each plot, excluding a 0.3‐m boundary zone at the edge of each plot. We used eight previously established permanent 10 × 10 cm quadrats (Fridley *et al*., 2011) and four newly established quadrats within each plot to guide sampling. Leaf tissue was recovered from the individual that was rooted most closely to the centre of each quadrat (12 samples per plot in total). The spatial locations of both the centre of each quadrat and of the sampled plant relative to this point were recorded to the nearest 5 mm. Soil depth at the 240 existing quadrats was known (Fridley *et al*., 2011). Local soil depths were determined for our newly established quadrats and for any plants rooted outside quadrats (i.e. for plants that were located > 5 cm from the quadrat centre). Our sampling scheme led to a total of 60 samples per climate treatment subpopulation. Samples from each treatment spanned the entire range of soil depths occupied by our study species at the site. Each leaf tissue sample was stored in self‐indicating silica gel (1–3 mm grain size; Merck‐Millipore, Darmstadt, Germany) for genetic analysis. Replicate tissue samples were collected from 40 randomly selected individuals to estimate genotyping error.

### Molecular methods

Laboratory protocols for DNA extraction from leaf tissue samples and subsequent AFLP analysis followed Whitlock *et al*. (2008a). Modifications and further details, including methods for quality control, are given in Text S2. AFLP peak‐height data for each individual were generated using genemapper version 3.0 (Applied Biosystems), and AFLP phenotypes were scored using aflpscore (Whitlock *et al*., 2008b; see Table S3 for phenotype scoring results and error rates). AFLP band presence–absence data for our study species comprised 270 loci and 222 individuals for *P. lanceolata*, and 999 loci and 303 individuals for *F. ovina*.

### Population density and abundance estimates

We estimated population density in order to understand whether the climatic environments at BCCIL had led to demographic changes that could have driven changes in genetic structure. Closest individual density estimation was used to assess population density for each study species, exploiting the known distance between the centres of the 100 cm^2^ quadrats and the nearest individual of each of our study species (Cottam *et al*., 1953; Cottam & Curtis, 1956; Text S2). Population density at BCCIL can be used as a proxy for population size on the basis that each of the plots and each of the treatments have identical areas. Existing species abundance data, collected for 240 of the quadrats, and measured as ordinal vegetation cover class (0 = 0–4%; 1 = 5–24%; 2 = 25–49%; 3 = 50–74%; 4 = 75% +; Fridley *et al*., 2011) were used to create plot‐level and treatment‐level summaries of species abundance. This cover‐based measure of species abundance is a function of both local plant density and plant morphology.

### Genetic diversity and spatial structure

Allele frequencies were calculated using Zhivotovsky's Bayesian method (1999) assuming Hardy–Weinberg equilibrium (R‐functions to carry out population genetic analyses are available as Files S10 and S11). Allele frequency estimates for globally polymorphic loci (0.05 ≤ *q* ≤ 0.95) were used to calculate gene diversity (an unbiased estimate of the expected heterozygosity) for climatic and soil‐depth subpopulations at BCCIL for each study species (Lynch & Milligan, 1994; Text S2).

We tested for the presence of spatial genetic structure (SGS) within each species by regressing pairwise kinship coefficients on the distance separating plant individuals (implemented using the software spagedi version 1.4; Hardy & Vekemans, 2002). Any loci identified as showing outlier patterns of differentiation among climate treatments under the analyses described below (i.e. those loci putatively responding to climatic selection) were removed prior to the assessment of SGS (*F. ovina*, 48 loci removed; *P. lanceolata*, 25 loci removed). Significance of the regression slope was tested using 2000 permutations of AFLP genotypes among sampling locations. The extent of SGS was estimated by quantifying Wright's neighbourhood size, which measures the effective population size of locally panmictic units within the total population, and *σ* (the gene dispersal parameter), which defines the physical size of these panmictic units (equal to half the expected parent–offspring dispersal distance). These analyses assume that populations have reached drift–dispersal equilibrium and that dispersal is isotropic and also require an estimate of effective population density (methods described in Text S2). Populations were assumed to be in Hardy–Weinberg equilibrium (i.e. *F*_IS_ = 0) on the basis of previous isozyme studies carried out on our study species (Bos *et al*., 1986; Weibull *et al*., 1991).

### Climate‐induced genetic differentiation

Genomewide (multilocus) differentiation between climatic environments was assessed using Cockerham & Weir's (1993) *β* estimator of *F*_ST_ (R‐functions given as Supporting Information). The significance of *F*_ST_ was determined by creating a null distribution for *F*_ST_ via 5000 permutations of individuals among climatic subpopulations (incorporating the observed *F*_ST_ as a datum). We used permutational analysis of variance to determine whether genetic distances among individuals could be explained by climate treatment, soil depth, treatment × soil depth interaction or block, using function ‘adonis’ in r package vegan 1.17‐4 (Legendre & Anderson, 1999; McArdle & Anderson, 2001). Permutational anova is an alternative to amova (Excoffier *et al*., 1992) that provides a flexible approach to partitioning variation in a distance matrix, allowing for continuously distributed explanatory variables, interaction terms and nonindependence due to grouping factors (incorporated by restricting permutations within different ‘strata’). Permutational anova analyses were conducted on Euclidian genetic distance matrices computed from the AFLP presence–absence data. First we carried out a global permutational anova analysis that included all treatment and control climates, in which a treatment × soil depth interaction and block main effects were fitted (*P*‐values for treatment effects were identical whether or not blocks were fitted as strata). Second, we performed additional analyses that paired each climate treatment with control. In these permutational anova, we fitted a treatment × soil depth interaction and incorporated blocks as strata. All of these analyses used 1999 permutations of the genetic distance matrix to compute significance tests on the parameter estimates.

To determine whether genetic structure was associated with local demographic history, we carried out a regression of plot‐level gene diversity (expected heterozygosity) against plot‐level harmonic mean subpopulation abundance over the 15 years of monitoring at BCCIL and against current subpopulation density. These former abundance data are point quadrat records drawn from Grime *et al*. (2008; Fig. S1); population abundance and population density are correlated in this system (see results). Genetic drift is expected to lead to a loss of genetic diversity that is related to local subpopulation size (Crow & Kimura, 1970). Thus, a positive relationship between local subpopulation size and genetic diversity would provide evidence in support of a role for local demographic isolation and genetic drift (neutral genetic change) in driving population structure at BCCIL. Harmonic mean abundance was used as a predictor for genetic diversity because the effects of drift on diversity in a temporally fluctuating population are expected to be dictated largely by periods of low effective population size (Wright, 1931; Crow, 1964). These analyses assume that census population sizes or abundance estimates are proportional to effective population sizes.

We tested for the genomic signature of adaptive differentiation in response to simulated climate change and soil‐depth heterogeneity using two outlier analyses. First, we conducted analyses using bayescan v. 2.1 (Foll & Gaggiotti, 2008). This software identifies individual marker loci showing patterns of differentiation consistent with departures from neutrality by modelling a parameter for neutral differentiation shared by all loci, and locus‐specific parameters for differentiation representing the effects of selection. For each locus, the weight of evidence in favour of a response to selection is calculated as an odds ratio of posterior probabilities for models containing either both the parameters or containing only the parameter for neutral differentiation. We ran bayescan models with a burn‐in period of 50 000 iterations and then extracted samples of size 1000 from MCMC chains, with a thin interval of 1000 iterations (1.05 × 10^6^ iterations in total). The prior odds for the neutral model were set to 10. We set a beta prior for *F*_IS_ with mean 0.05 and standard deviation 0.1 to incorporate information regarding the mating system of our study species (Bos *et al*., 1986; Weibull *et al*., 1991). This prior incorporates previous evidence for a value of *F*_IS_ close to zero, but allows for uncertainty in the precise value and for nonzero *F*_IS_ in our study populations. Loci responding to selection were identified when the posterior odds for a response to selection exceeded three (equivalent to ‘substantial’ support for selection as defined by Jeffrey's scale of evidence for Bayes factors; Jeffreys, 1961). We tested for responses to climatic selection by undertaking a global analysis including all climatic environments and by constructing pairwise outlier analyses for each combination of climate treatment with control, pooling individuals across plots within treatments (five analyses, each with treatment and control subpopulations). Plot‐level sample sizes (*n* = 12) were insufficient to support separate, robust outlier analyses for each climate treatment within each block. We also carried out a bayescan analysis that used experimental blocks as subpopulations, to test for signatures of selection resulting from spatial variation in the environment at BCCIL. The second outlier analysis used logistic regression to investigate associations between the presence–absence phenotypes at individual AFLP loci and climate treatment and soil depth (soil depth could not be modelled in the bayescan analysis). Logistic regressions were fitted separately for each locus, using the R function bayesglm, with a logit link (package arm; Gelman *et al*., 2009). For each locus, we fitted a treatment × soil depth interaction and block main effect. This model was reduced sequentially, and likelihood ratio tests were used to assess the treatment × soil depth interaction, and the treatment, soil depth and block main effects. To identify loci putatively responding to environmental selection, we estimated the tail area‐based false discovery rate for each locus within each model effect, using the total set of *P*‐values across loci within model effects (implemented in R package fdrtool; Strimmer, 2008a,b). Model effects for particular loci were considered to show an outlier pattern of genetic differentiation when their corresponding *Q*‐value was below 10%.

### Genetic vs. community‐level responses to climate change

We carried out two analyses in order to determine whether responses to simulated climate change at BCCIL at the community level were coupled with multilocus genetic responses within species. First, we performed correlations of plot‐level species diversity (species richness and Shannon diversity index) with multilocus gene diversity [species abundance data were taken from Fridley *et al*. (2011)]. Second, we calculated the ecological distance between all pairs of plots using the Bray–Curtis index and compared the resulting dissimilarity matrix with a matrix of multilocus pairwise genetic distances (*F*_ST_), using a simple mantel test.

## Results

### Population structure and diversity

**Figure 3:**
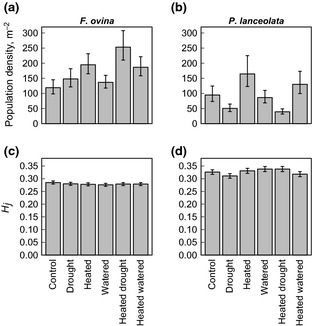
Population density and gene diversity (expected heterozygosity) of subpopulations of *F. ovina* and *P. lanceolata* growing in different climatic environments at BCCIL. (a, b) Population density estimated from the distance between the centre of each 10 × 10 cm permanent quadrat and the nearest (sampled) individual of *F. ovina* or *P. lanceolata*. (c, d) Gene diversity estimates (*H*_j_) based on polymorphic AFLP loci (613 loci, *F. ovina*; 221 loci, *P. lanceolata*). Estimates of *H*_j_ were based on identical sample sizes for each climate environment subpopulation (Text S2). Error bars show one standard error of the mean.

Population density varied significantly with climate change treatment for both study species (*F. ovina*:* F*_5,295_ = 4.3, *P* = 0.046; *P. lanceolata*:* F*_5,213_ = 18.8, *P* < 0.001; Fig. 3a, b). Both study species responded positively to the warming treatment, but showed opposing responses to the drought treatment (increase in rooted plant density in *F. ovina*, decrease in *P. lanceolata*; Fig. 3a, b). Both species also exhibited a pattern of increasing plant density with soil depth (*P* < 0.05 in each case). Plot‐level estimates of population density were associated positively with plot‐level abundance (cover) within the permanent quadrats (*F. ovina*:* r* ^2^ = 0.27, *P* = 0.007; *P. lanceolata*:* r* ^2^ = 0.69, *P* < 0.001).

*Festuca ovina* and *P. lanceolata* populations occupying intact limestone grassland at BCCIL were genetically diverse; gene diversity estimates (*H*_j_) based on polymorphic AFLP loci were 0.281 and 0.332 for *F. ovina* and *P. lanceolata*, respectively. All individuals possessed a unique multilocus AFLP genotype, indicating that all sampled plants represented distinct genetic individuals. Subpopulations of *F. ovina* that had been exposed for 15 years to different simulated climatic environments possessed closely similar gene diversities (Fig. 3c). Standard error estimates for gene diversity in *P. lanceolata* indicated weak variation in diversity among climate treatment subpopulations (Fig. 3d). Drought‐treated subpopulations of *P. lanceolata* had the lowest genetic diversity (*H*_j_ = 0.311), and watered and heated‐drought subpopulations were the most genetically diverse (*H*_j_ = 0.338; Fig. 3d). Levels of genetic diversity within these species did not vary consistently along the soil‐depth gradient present at the study site (between shallow and deep soils; Table S4).

Both study species showed evidence of spatial genetic structure (regression slopes of pairwise kinship coefficients on distance were significantly negative *P* < 0.001). Neighbourhood sizes (the effective size of locally panmictic units within the population) were 320 and 140, and neighbourhood areas were 9.29 and 9.97 m^2^ for *F. ovina* and *P. lanceolata*, respectively (assuming an *N*_E_/*N* ratio of 50%). These neighbourhood areas are approximately the size of individual plots at BCCIL (9 m^2^). Neighbourhood sizes and areas were substantially greater for *F. ovina* when we assumed an *N*_E_/*N* ratio of 10% (636 individuals and 70.68 m^2^, respectively). The procedure used to estimate dispersal parameters did not converge using an assumed *N*_E_/*N* ratio of 10% when applied to *P. lanceolata*.

### Genetic differentiation

Subpopulations of both study species showed weak but significant genetic differentiation among the climatic environments at BCCIL (*F. ovina*:* F*_ST_ = 0.006, permutation test *P* < 0.001; *P. lanceolata*:* F*_ST_ = 0.012, *P* < 0.001; Table S5). Permutational anova analysis supported the presence of climate‐associated differentiation (partial *R* ^2^ = 0.02 and 0.03 for *F. ovina* and *P. lanceolata*, respectively; *P* < 0.001; Table 1) and also indicated significant genetic differentiation among experimental blocks (partial *R* ^2^ = 0.02 and 0.03; *P* < 0.001; Table 1). The majority of genetic variance in these analyses was unstructured with respect to both experimental treatments and experimental blocks (partial *R* ^2^ for residual variance = 0.94 and 0.91; Table 1). There were weak associations between the genetic distance between individuals and soil depth, for both *F. ovina* (*R* ^2^ = 0.004, *P* = 0.018) and *P. lanceolata* (*R* ^2^ = 0.005, *P* = 0.057; Table 1). Pairwise treatment–control comparisons for *F. ovina* revealed significant climate‐induced genetic differentiation for all climatic environments relative to control (results for drought‐treated and watered plots remained significant following Bonferroni correction; Table 1). *Plantago lanceolata* subpopulations showed significant climate‐induced genetic differentiation in all cases except the heated–control contrast. Only the watered–control pairwise contrast remained significant following Bonferroni correction (Table 1). The greatest genetic distances (*F*_ST_) between individual climate treatments and control plots occurred for the watered treatment (*F*_ST_ = 0.007, *F. ovina*;* F*_ST_ = 0.016, *P. lanceolata*; pairwise *F*_ST_ matrices are given in Table S6). There was no clear correspondence between the occurrence of control–climate treatment genetic differentiation (Table 1) and differences in subpopulation density (Fig. 3a, b). For example, in *P. lanceolata,* we observed no genetic differentiation of the heated (winter warming) from control subpopulations (Table 1), yet winter warming had modified this species’ density substantially (73.6% increase in density; Fig. 3b). Both drought‐treated and watered subpopulations of *F. ovina* were significantly differentiated from the control subpopulation (Table 1), but the drought and watering treatments elicited only modest changes in population density (increase in density < 25%; Fig. 3a).

**Table 1:** Permutational anova analyses show that subpopulations of *Festuca ovina* and *Plantago lanceolata* exposed to 15 years of simulated climate change have become genetically differentiated. Permutational anova analyses were carried out on Euclidian distance matrices derived from multilocus AFLP genotypes for sampled individuals. Results are given for models that included all treatments and for models that used individual pairwise treatment–control comparisons. All analyses following the global analysis were stratified by experimental block, and so block terms are not shown for these analyses

	*Festuca ovina*					*Plantago lanceolata*				
	df	SS	*F*	*R* ^2^	§ *Pr* (> F)	df	SS	*F*	*R* ^2^	§ *Pr* (> F)
All treatments										
Treatment	5	507	1.23	0.020	0.0005***	5	210	1.32	0.030	0.0005***
Depth	1	96	1.17	0.004	0.0175*	1	38	1.20	0.005	0.0565†
Block	4	453	1.37	0.018	0.0005***	4	226	1.78	0.032	0.0005***
Treatment × Depth	5	425	1.03	0.017	0.1735	5	171	1.08	0.024	0.0865†
Residuals	287	23680		0.941		204	6472		0.909	
Total	302	25162		1.000		219	7117		1.000	
Drought–Control										
Treatment	1	108	1.28	0.018	0.0005**	1	40	1.29	0.019	0.0090
Depth	1	95	1.14	0.016	0.1330	1	30	0.96	0.014	0.5710
Treatment × Depth	1	75	0.90	0.013	0.9450	1	34	1.07	0.016	0.2775
Residuals	68	5696		0.953		64	2004		0.951	
Total	71	5974		1.000		67	2108		1.000	
Heated–Control										
Treatment	1	99	1.19	0.013	0.0270	1	37	1.14	0.016	0.1010
Depth	1	93	1.12	0.012	0.3255	1	36	1.13	0.016	0.2740
Treatment × Depth	1	91	1.09	0.012	0.1815	1	31	0.96	0.014	0.7505
Residuals	91	7563		0.964		68	2185		0.955	
Total	94	7845		1.000		71	2289		1.000	
Watered–Control										
Treatment	1	110	1.32	0.014	0.001*	1	44	1.33	0.017	0.0035†
Depth	1	99	1.19	0.012	0.0470	1	36	1.10	0.014	0.4080
Treatment × Depth	1	86	1.03	0.011	0.3655	1	27	0.81	0.010	0.9745
Residuals	92	7625		0.963		75	2473		0.958	
Total	95	7919		1.000		78	2580		1.000	
Heated‐drought–Control										
Treatment	1	103	1.23	0.014	0.0185	1	43	1.34	0.019	0.0055†
Depth	1	92	1.11	0.012	0.4560	1	32	1.00	0.014	0.5265
Treatment × Depth	1	93	1.11	0.013	0.1225	1	38	1.18	0.017	0.0970
Residuals	85	7094		0.961		67	2176		0.950	
Total	88	7381		1.000		70	2290		1.000	
Heated‐watered–Control										
Treatment	1	100	1.19	0.013	0.0245	1	43	1.37	0.019	0.002*
Depth	1	94	1.12	0.012	0.3055	1	37	1.16	0.016	0.1015
Treatment × Depth	1	80	0.96	0.010	0.7295	1	31	0.97	0.013	0.5365
Residuals	91	7602		0.965		70	2207		0.952	
Total	94	7876		1.000		73	2317		1.000	

§*P*‐values are given as raw (unadjusted) values.

***,**,*,†Indicate that Bonferroni‐adjusted *P*‐values were < 0.001, < 0.01, < 0.05 or < 0.10, respectively. No Bonferroni correction was applied to the models that included all treatments.

To determine whether the data supported a role for drift in shaping genetic structure, we regressed plot‐level gene diversity on ‘long‐term’ (15‐year) population abundance estimates and on current population density. Gene diversity was associated with neither population abundance (*F. ovina*:* R* ^2^ = 0.010, *P* = 0.625; *P. lanceolata*:* R* ^2^ = 0.039, *P* = 0.293) nor current population density (*F. ovina*:* R* ^2^ = 0.005, *P* = 0.725; *P. lanceolata*:* R* ^2^ = 0.019, *P* = 0.468).

### Response to climatic selection

Outlier analyses using bayescan failed to detect any outlier loci associated with experimental climatic environment for *F. ovina* (*Q*‐values were all > 0.5; posterior probabilities for a response to selection were all < 0.5). Two of the AFLP loci typed in *P. lanceolata* showed evidence of genetic divergence in response to climatic selection (pc1_ned_60: *Q*‐value = 0.124, posterior probability = 0.876, *F*_ST_ (Cockerham & Weir, 1993) = 0.201; pc1_fam_55: *Q*‐value = 0.126, posterior probability = 0.873, *F*_ST_ = 0.218). These loci were identified in the analysis involving all climate treatment subpopulations.

Logistic regression identified two *F. ovina* AFLP loci (X306.3_TCT_CGG_Fo and X327_TCC_CGC_Fo) with significant variation in fragment frequency between climate change treatments (*F*_ST_ = 0.036 and 0.030, respectively). Similar analyses identified eighteen *P. lanceolata* loci as showing outlier patterns of genetic differentiation among climate change treatments, which included the two previously identified outlier loci for this species (range in *F*_ST_ = 0.021–0.218). Soil depth and the soil depth × treatment interaction were not significant predictors of AFLP fragment frequency for any locus in either study species. Genetic differentiation among climate treatments at putatively neutral (nonoutlier) loci was low for both species (*F. ovina,* median *F*_ST_ = 0.003; *P. lanceolata*, median *F*_ST_ = 0.003).

### Outlier loci associated with experimental block

Outlier analysis using bayescan identified eight *P. lanceolata* loci that showed significant divergence among experimental blocks (i.e. with naturally occurring spatial variation in the block environment; all *Q*‐values < 0.1, posterior probability for a response to selection = 0.744–0.999). The same eight loci were present in the sets of candidate outliers identified by analysis of the block environment using logistic regression. In contrast, analysis of the block environment using bayescan did not detect any *F. ovina* AFLP locus with an outlier pattern of genetic differentiation (all *Q*‐values ≥ 0.350).

### Community‐level vs. genetic structure

Correlations between species‐level and genetic diversity were all small and nonsignificant for both study species (¦ *r*_P_ ¦ < 0.1; *P* > 0.05). Similarly, associations between species‐level ecological dissimilarity and genetic differentiation within species were weak and nonsignificant (*F. ovina*:* r*_M_ = 0.174, *P* = 0.071; *P. lanceolata*:* r*_M_ = 0.086, *P* = 0.225).

## Discussion

We investigated species' genetic responses to 15 years of simulated climate change applied to intact species‐rich limestone grassland, focusing on two of the most abundant species within the community. Our aims were to understand the extent to which genetic responses to the climate are possible within grassland plants, and whether these responses are consistent with a process of evolution in response to climatic selection. We detected genetic differentiation among long‐term climate treatments at BCCIL using both genomewide multilocus analyses (*F*_ST_, permutational anova) and single locus‐oriented outlier analyses. This differentiation has occurred within contiguous grassland over a very small spatial extent (thirty 9‐m^2^ plots spread over approximately 0.2 ha grassland), and happened rapidly, during 15 years of climate change manipulations. Similarly, rapid genetic change occurring in response to a natural drought has been documented in the annual species *Brassica rapa* (Franks *et al*., 2007). Treatment‐level genetic structure at BCCIL is unlikely to be dominated by neutral spatial genetic structuring because each treatment is replicated in each spatial experimental block. Furthermore, we detected two outlier AFLP loci associated with climate treatment, which possessed patterns of differentiation that were statistically distinct from the background of putatively neutral genomic differentiation. This signature of climatic selection may reflect climate‐driven adaptive evolutionary change within populations at BCCIL.

Our survey data suggested that 15 years of experimental climate change had altered population density within some of the experimental treatments (in particular, the drought, heated and heated‐drought treatments; Fig. 3a, b), indicating modified patterns of survival and recruitment within these environments. These changes are known to have altered community structure at fine spatial scales (Fridley *et al*., 2011). Such demographic responses may be sufficient to induce neutral genetic change through drift and the loss of genetic diversity at low population size, assuming that subpopulations at BCCIL are isolated demographically. Our data, however, did not provide evidence in support of this effect. Levels of gene diversity within individual plots were not associated with either (long‐term) estimates of population abundance or population density, for either species. Furthermore, although we found evidence in both species for genetic differentiation in all treatments except the winter warming treatment (permutational anova analysis), there was no obvious link between the extent or presence of genetic differentiation and treatment‐mediated changes in population density. Hence, neither genetic diversity nor genetic differentiation was associated consistently with sustained changes in population size at BCCIL.

Effective gene flow would be expected to homogenize the distribution of genetic variation at selectively neutral loci, limiting the effects of drift within subpopulations at BCCIL (Wright, 1931). Such gene flow may be especially likely in our study species, which produce wind‐dispersed pollen and are highly outcrossing (Morjan & Rieseberg, 2004). Analyses of fine‐scale spatial genetic structure were consistent with panmixia at putatively neutral AFLP loci at, or exceeding, the scale of the experimental blocks at BCCIL (~9 m^2^; each climate treatment is represented in every block). Cross‐fertilization between plant populations can occur at appreciable rates at scales of 100–1000 m (Ellstrand, 2014), and thus, the spatial scale of the experiment at BCCIL is unlikely to pose a problem for gamete (pollen) movement among climate change treatments. Moreover, the low levels of genetic differentiation observed among the climate treatments at putatively neutral (nonoutlier) AFLP loci (median *F*_ST_ = 0.003) are also consistent with a scenario of effective gene flow. *F*_ST_ can be used to calculate a crude measure of the effective number of migrants per generation (*N*_e_ *m*) exchanged between populations (Wright, 1931). Observed values of *F*_ST_ between climate change treatment pairs at BCCIL imply *N*_e_ *m* > 10 for all treatment pairs in either species (see Whitlock & McCauley, 1999 for the limitations of this approach). Additional analyses using the software structure (Pritchard *et al*., 2000; Text S7, Figures S8 and S9) provide further support for the prevalence of gene flow, indicating only very weak genetic structure that was not associated with simulated climate change treatments. Finally, recent studies of floral phenology suggest substantial overlaps in flowering time between subpopulations occupying different climate treatments at BCCIL (S. Buckland, unpublished data). Thus, we have no reason to believe that any barrier to gene flow has arisen through asynchrony in floral timing, which could otherwise have facilitated genetic drift (Fox, 2003; Hendry & Day, 2005). We conclude that it is extremely unlikely that climatic subpopulations at BCCIL have become demographically isolated from each other. Hence, it also seems unlikely that genetic drift has had a marked impact on treatment‐level genetic structure.

Outlier analyses detected a consistent signature of selection in response to simulated climate change at individual AFLP loci in *P. lanceolata*. Two outlier loci, identified in both outlier analyses, showed genetic differentiation (*F*_ST_ ≈ 0.2) greatly exceeding the average background genetic differentiation at putatively neutral loci in this species (median *F*_ST_ = 0.003). Genome regions associated with the two outlier loci identified consistently in *P. lanceolata* represent candidate targets for climatic selection and may control phenotypes that are adaptive under the modified climates at BCCIL. The observed genetic differentiation at the putatively selected AFLP loci occurred against a background of low genomewide differentiation. As we argue above, low levels of background differentiation are likely to have been maintained by gene exchange among grassland plots. Thus, implying that climatic selection has had to work against gene flow to establish divergence at the genome regions putatively responding to selection.

Evidence for a response to climatic selection in *F. ovina* was equivocal; outlier loci associated with climate change treatment were only detected using logistic regression and were weakly differentiated (*F*_ST_ ≈ 0.03; neutral background differentiation = 0.003). Failure to detect a stronger response to climatic selection in *F. ovina* may simply represent the absence of a response, but it may also reflect a genomic marker density insufficient to detect such an effect. In other words, linkage disequilibrium (LD), which may create associations between loci responding to climatic selection and neutral loci, may decay too rapidly for our genotyping strategy to be useful. For example, in maize, LD can decay within 10 kb (Flint‐Garcia *et al*., 2003; Yan *et al*., 2009), and coalescence simulations suggest that, in outbreeding species, LD can decay completely within 4 kb (Nordborg, 2000). The 2C DNA values for *F. ovina* (Grime *et al*., 1985) indicate that the AFLP markers will be spaced approximately every 4.7 Mb on average (assuming 978 Mb per picogram DNA).

The argument for a climatically adaptive response to selection is supported by recent common environment experiments using plants collected from BCCIL documenting climate‐associated evolutionary changes in phenotype in both of our study species (Ravenscroft *et al*., 2014; R. Whitlock, unpublished data). For example, when grown in a common environment, *P. lanceolata* individuals from the drought plots at BCCIL make a greater investment in reproductive output, whereas those from control plots allocate more resources to vegetative growth (Ravenscroft *et al*., 2014). The expression of differentiated phenotypes under a common environment demonstrates a genetic basis to phenotypic differentiation. Thus, these studies show that populations of grassland plants at BCCIL are capable of evolutionary responses to climatic selection.

How do the community‐level responses to simulated climate change documented previously at BCCIL contrast with impacts below the species‐level documented in this study, and are these outcomes correlated? Species abundance and community composition at BCCIL have been shown to be mediated through the effects of both climate treatment and soil depth; these factors had comparable effect sizes on plant community structure (Fridley *et al*., 2011). In contrast, multilocus (genomewide) genetic responses to simulated climate change were dominated by treatment‐level effects; the effects of soil depth were at least fivefold smaller in magnitude than those attributable to treatment (Table 1). Furthermore, there was no clear relationship between the extent of community‐level compositional shifts in different climate treatments and the strength of genetic differentiation within individual species. For example, the highest multilocus genetic differentiation (*F*_ST_) was observed for the watered treatment, yet this was the treatment with the lowest community‐level compositional change relative to control plots (Fridley *et al*., 2011). Finally, we found no evidence to support relationships between species diversity and multilocus genetic diversity or between ecological distance (community dissimilarity) and multilocus genetic distance within species. Thus, genomewide genetic responses to simulated climate change within individual species were likely to have been decoupled from, or occurring independently of community‐level compositional responses. We predict, however, that the abundance of individual species at BCCIL will be, in part, a function of genetic structure at a subset of genomic loci (including outlier loci) that mediate adaptation to particular climatic environments.

Our results are novel in demonstrating rapid genetic divergence in the established (reproductively mature) phase of perennial plant species in an intact ecosystem in response to a suite of simulated climate change environments. As the climate treatments are replicated in a spatially blocked experimental design (Grime *et al*., 2000), it seems likely that this divergence has arisen specifically in response to the climate treatments themselves, in parallel with community‐level compositional change (Fridley *et al*., 2011). Furthermore, genetic divergence has occurred over a relatively small period of evolutionary time (15 years of experimentation is ≤ 15 generations for our study species). Our findings are consistent with a scenario in which climate‐induced selection imposed at BCCIL has driven climatically adaptive genetic divergence, against a background of weak differentiation maintained by gene flow. However, it is important to note that, based on the available data, we cannot exclude the possibility that genetic drift has contributed to differentiation among climate change treatments at BCCIL. Future work at BCCIL will focus on verifying and functionally characterizing the genomic signature of climatic selection documented in this study and will seek to determine the relationship between climatically selected genotypes, climatically adaptive phenotypes and species abundance.

## Data accessibility

AFLP genotypes, spatial data and sample information (including experimental treatment, block and soil depth at sampling site): DRYAD entries.

## Supporting information


**Figure S1.** Changes in abundance of *Festuca ovina* and *Plantago lanceolata* during the first 15 years of climatic manipulations at BCCIL.
**Text S2.** Detailed methods for field survey and sample collection, AFLP genotyping and population genetics analysis.
**Table S3.** Selective primer combinations used in the AFLP analysis and results for AFLP phenotype scoring.
**Table S4.** Diversity statistics for climatic sub‐populations of *F. ovina* and *P lanceolata* at BCCIL.
**Table S5.** Genetic differentiation among climatic and edaphic sub‐populations of *F. ovina* and *P. lanceolata* at BCCIL.
**Table S6.** Pairwise genetic differentiation between climate treatment sub‐populations of *F. ovina* and *P. lanceolata* at BCCIL.
**Text S7.** structure analysis.
**Figure S8.** Graphical output from structure harvester.
**Figure S9.** structure plots.

**File S10.** R‐script containing functions for population genetics analyses.

**File S11.** Readme file for R functions.
