# Supplementary material for: Rapid genetic divergence in response to 15 years of simulated climate change
Source: Glob Chang Biol. 2015 Aug 27;21(11):4165–76. doi: 10.1111/gcb.12966 (PMC4975715; doi:10.1111/gcb.12966)
Supplement: Supplementary file 1 — Figure S1. Changes in abundance of Festuca ovina and Plantago lanceolata during the first 15 years of climatic manipulations at BCCIL. Text S2. Detailed methods for field survey and sample collection, AFLP genotyping and population genetics analysis. Table S3. Selective primer combinations used in the AFLP analysis and results for AFLP phenotype scoring. Table S4. Diversity statistics for climatic sub‐populations of F. ovina and P lanceolata at BCCIL. Table S5. Genetic differentiation among climatic and edaphic sub‐populations of F. ovina and P. lanceolata at BCCIL. Table S6. Pairwise genetic differentiation between climate treatment sub‐populations of F. ovina and P. lanceolata at BCCIL. Text S7. structure analysis. Figure S8. Graphical output from structure harvester. Figure S9. structure plots. [file GCB-21-4165-s001.docx]

**Figure S1** Changes in abundance of *Festuca ovina* and *Plantago lanceolata* during the first 15 years of climatic manipulations at Buxton Climate Change Impacts Lab (BCCIL). Each point is a mean value of abundance estimated from plot-level (3 × 3 m) point quadrat surveys carried out in five replicate plots ([data from Grime *et al.*, 2008](#_ENREF_5)). Error bars represent one standard error of the mean. Points for each year have been jittered along the x-axis to facilitate identification of points and error bars.

**Text S2** Detailed methods for field survey and sample collection, AFLP genotyping and population genetics analysis

*Determining spatial locations for sampled plants*

The centre of each 10 × 10 cm quadrat at BCCIL is marked permanently with a labelled metal pin. Tape measures were used to derive relative *x* and *y* coordinates for each quadrat pin relative to the “origin” (bottom left-hand corner) of each grassland plot (to the nearest cm). Steel rulers were then used to measure the *x* and *y* displacements of the nearest individuals of our study species to the quadrat centre (metal pin), to the nearest 0.5 cm. In addition we determined the relative positions and alignments of neighbouring plots to each other, using tape measures and compass bearings. This process allowed us to calculate relative spatial locations for all sampled plants and for all quadrat centres. Finally, we anchored our spatial data on the British Ordnance Survey National Grid System (OSGB 36), using a handheld GPS unit accurate to 10 m. This allowed us to correct cumulative distortions of the plot map over the BCCIL site, and to express the location of plants and quadrats on the OSGB 36 system (i.e. relative to the origin of this grid system). It is important to note that the accuracy of distances measured on this spatial framework is likely to decline as distance increases; displacements measured at the finest spatial scales will be the most accurate (with up to 0.5 cm accuracy).

*Soil depth measurement*

Soil depths for newly established quadrats, or for plants located outside of quadrats were determined using a 10 mm aluminium soil depth probe driven by hand down to the limestone bedrock. Quadrat soil depths were taken to be the mean of five soil depth measurements taken at the corners and the centre of the quadrat.

*Plant density estimation*

Plant density was determined using known Euclidean distances between sampling locations (centres of permanent quadrats) and sampled plants. We derived density estimates for climate treatment sub-populations by applying a square root transformation to the raw distance data (*x*). A linear model was applied to these transformed data with climate treatment and (centred) soil depth predictors. Model predictions and error intervals were converted from distances to densities by back-transforming, and then applying the following formula: *d = 1 / 4 ^2^* , where is an estimate of mean distance between the quadrat centres and the nearest plant individuals. Other (plot-level and experiment-wide) estimates of population density were derived by setting to the respective arithmetic mean plant–sampling location distance.

Global (experiment-wide) population density estimates were used to determine effective population density for each study species under the assumption that effective population sizes are typically 10–50% of census population sizes ([Frankham, 1995](#_ENREF_4), [Palstra & Ruzzante, 2008](#_ENREF_9)). Thus, two values of *N*_E_/*N* were used: 50% and 10%.

*Plant tissue collection*

Plant tissue samples were collected in 1.5-ml screw-cap tubes (Sarstedt Art. No. 72.692.105) containing approximately 1.2 ml desiccating silica gel (1–3 mm grain size; Merck). Sample tubes were labelled with time tape (TimeMed Labelling Systems). The samples were stored at room temperature until DNA extraction.

*AFLP genotyping and quality control*

We used a plate centrifuge for DNA elution steps 1–4 described in Whitlock *et al*. (2008a). Pre-selective PCR amplification products were diluted to a factor of 1:12 and 1 μL of this diluted product was used as a template for selective amplification. No formamide was added to the selective amplification mixture and selective amplification products were diluted by a factor of 1:25. Diluted PCR products (1 μL) were added to 9 μL of size standard mix containing 5 μL ROX 500 size standard (Applied Biosystems), 2 μL of ROX 585 size standard (to increase read length) and 1 ml HiDi formamide (Applied Biosystems). Fragments were separated using an ABI 3730 capillary sequencer (Applied Biosystems). These modifications produced the most consistent and clear AFLP profiles based on test runs using 48 individuals. Eight primer combinations were selected for each species from a test panel of 32 based on clarity of the AFLP profiles. For each species, sample positions were randomized across four 96-well plates, and each plate included two positive (leaf material from the same individual) and two negative controls (blank sample with no leaf material added). Replicate tissue samples (40) were run on a separate plate.

AFLP chromatograms were analysed using the software GENEMAPPER version 3.0 (Applied Biosystems). Sizing quality was checked manually for each fingerprint and samples with size standards of insufficient quality were rejected from the analysis. Bins were created automatically in GENEMAPPER. Bin positions were checked manually to ensure positions had been assigned correctly. Bins that were off centre were manually adjusted to centre on peaks. Bins that included fragments with wide peak morphology and bins that included fragments that were continuous with other bins were rejected from the analysis because of the potential for size homoplasy. Weak fingerprints with low peak height or short read length were rejected. Fingerprints that contributed many unique peaks were rejected from the analysis, as this could be a result of contamination. It was necessary to exclude *Festuca ovina* AFLP fingerprints originating from the control and drought-treated plots of blocks B and E, due to poor fingerprint quality. Therefore, in *Festuca ovina*, these treatments were represented by only three plots each (from blocks A, C and D).

*Gene diversity estimation*

Allele frequencies were calculated using Zhivotovsky’s Bayesian method ([1999; R-scripts have been provided as Supporting Information](#_ENREF_13)). We calculated gene diversity statistics using the approach given by Lynch & Milligan ([1994; see Supporting Information for R-scripts](#_ENREF_8)). Gene diversity estimates calculated in this way are sensitive to sample size. Therefore we resampled the sub-populations for which we estimated gene diversity to the sample size for the smallest sub-population. Samples were drawn randomly from within each sub-population without replacement. For each sub-population we used the mean of 50 resampled datasets to estimate gene diversity. We calculated gene diversity for climate treatment sub-populations, and for soil-depth sub-populations within each treatment, by splitting samples about their median rooted soil depths (creating “deep” and “shallow” soil depth classes within each treatment).

**Table S3** Selective primer combinations used in the AFLP analysis and results for AFLP phenotype scoring. The initial number of candidate loci output by genemapper is given, followed by the number of loci retained after phenotype scoring with aflpscore ([Whitlock *et al.*, 2008](#_ENREF_12)). The mismatch error rate is shown for retained loci and was calculated using AFLP chromatograms generated from 40 replicate tissue samples.

| Species | Primer Combination | Initial no. loci | Retained loci | Mismatch error rate % |
| --- | --- | --- | --- | --- |
| *Festuca ovina* | AGG-CAC | 131 | 114 | 5.6 |
|  | AGG-CAG | 128 | 126 | 5.1 |
|  | AGG-CTC | 132 | 124 | 5.2 |
|  | TCC-CCG | 146 | 125 | 5.7 |
|  | TCC-CGC | 162 | 150 | 5.4 |
|  | TCT-CCC | 88 | 78 | 3.6 |
|  | TCT-CGC | 134 | 122 | 2.5 |
|  | TCT-CGG | 168 | 160 | 3.0 |
|  | All | 1089 | 999 | 4.5 |
| *Plantago lanceolata* | TCC-CAA | 57 | 44 | 2.5 |
|  | TCC-CAT | 62 | 47 | 3.0 |
|  | TCC-CAG | 42 | 35 | 5.0 |
|  | TCC-CCT | 40 | 28 | 6.0 |
|  | TGA-CGA | 51 | 41 | 7.0 |
|  | TGA-CGT | 46 | 39 | 2.5 |
|  | TGA-CCA | 51 | 36 | 7.5 |
|  | TGA-CCC | 57 | 0 | (30.0)* |
|  | All | 406 | 270 | 4.6^§^ |

* This primer combination was not retained in the AFLP dataset for *P. lanceolata* due to its high error rate

§ The total error rate estimate for *P. lanceolata* excludes primer combination TGA-CCC

**Table S4** Diversity statistics for climate treatment sub-populations of *F. ovina* and *P. lanceolata* at BCCIL

|  |  | ***Festuca ovina*** | | | ***Plantago lanceolata*** | | |
| --- | --- | --- | --- | --- | --- | --- | --- |
| **Treatment** | **Soil depth sub-population** | **n** | **^*^PLP** | **^§^Gene diversity, Hj (s.e.)** | **n** | **^*^PLP** | **^§^Gene diversity, Hj (s.e.)** |
| All treatments | All | 303 | 61.4 | 0.281 (0.006) | 220 | 81.9 | 0.332 (0.009) |
|  | Shallow | 150 | 64.2 | 0.279 (0.006) | 110 | 83 | 0.327 (0.010) |
|  | Deep | 153 | 63.3 | 0.281 (0.006) | 110 | 87.4 | 0.331 (0.009) |
| Control | All | 36 | 63.5 | 0.285 (0.006) | 36 | 82.2 | 0.326 (0.009) |
|  | Shallow | 18 | 63.6 | 0.284 (0.006) | 18 | 83 | 0.309 (0.009) |
|  | Deep | 18 | 67.4 | 0.286 (0.005) | 18 | 87.4 | 0.341 (0.009) |
| Drought | All | 36 | 62.1 | 0.280 (0.006) | 32 | 82.6 | 0.311 (0.009) |
|  | Shallow | 18 | 63.3 | 0.282 (0.005) | 16 | 81.5 | 0.311 (0.009) |
|  | Deep | 18 | 66.2 | 0.279 (0.006) | 16 | 75.2 | 0.314 (0.009) |
| Heated | All | 59 | 63.2 | 0.278 (0.006) | 36 | 81.9 | 0.331 (0.010) |
|  | Shallow | 29 | 65.8 | 0.277 (0.006) | 18 | 82.6 | 0.334 (0.010) |
|  | Deep | 30 | 64.1 | 0.277 (0.006) | 18 | 79.3 | 0.321 (0.009) |
| Heated drought | All | 53 | 64.5 | 0.279 (0.006) | 35 | 81.9 | 0.338 (0.010) |
|  | Shallow | 26 | 62.5 | 0.279 (0.006) | 17 | 84.8 | 0.344 (0.010) |
|  | Deep | 27 | 60.9 | 0.276 (0.006) | 18 | 83.3 | 0.335 (0.010) |
| Heated watered | All | 59 | 63.3 | 0.279 (0.006) | 38 | 79.6 | 0.318 (0.010) |
|  | Shallow | 29 | 64.4 | 0.282 (0.006) | 19 | 80.4 | 0.318 (0.010) |
|  | Deep | 30 | 66.1 | 0.275 (0.006) | 19 | 83.7 | 0.312 (0.009) |
| Watered | All | 60 | 63.1 | 0.276 (0.006) | 43 | 85.9 | 0.338 (0.010) |
|  | Shallow | 30 | 64.7 | 0.273 (0.006) | 21 | 85.9 | 0.340 (0.010) |
|  | Deep | 30 | 65.9 | 0.280 (0.006) | 22 | 88.9 | 0.337 (0.010) |

* Percentage of loci polymorphic ([calculated using aflp-surv; Vekemans, 2002](#_ENREF_11))

§ Excludes monomorphic loci (0.05 ≤ allele frequency ≤ 0.95)

**Table S5** Genetic differentiation among climate treatment and edaphic sub-populations of *F. ovina* and *P. lanceolata* at BCCIL, measured using Cockerham and Weir’s *β* statistic ([1993; R-scripts provided as Supporting Information](#_ENREF_1)). The 2.5% and 97.5% quantiles give critical values for a null distribution of *β* obtained via 5000 permutations of individuals among sub-populations (the null distribution included the observed value of *β* as a datum). Soil-depth sub-populations (“deep” or “shallow”) were defined using the median soil depth as an arbitrary threshold for sub-population membership. *P*-value gives a two-sided *P*-value calculated by comparing the observed *β* statistic to the permuted null distribution.

| **Species** | **Sub-populations** | **No. sub-populations** | **Observed *β*** | **2.5% quantile** | **97.5% quantile** | ***P*-value** |
| --- | --- | --- | --- | --- | --- | --- |
| *F. ovina* | Soil depth | 2 | 0.0023 | 0.0015 | 0.0029 | 0.639 |
|  | Treatment | 6 | 0.0063 | 0.0030 | 0.0046 | < 0.001 |
|  | Treatment × soil depth | 12 | 0.0066 | 0.0028 | 0.0047 | < 0.001 |
| *P. lanceolata* | Soil depth | 2 | 0.0030 | 0.0008 | 0.0045 | 0.396 |
|  | Treatment | 6 | 0.0121 | 0.0029 | 0.0074 | < 0.001 |
|  | Treatment × soil depth | 12 | 0.0118 | 0.0029 | 0.0084 | < 0.001 |

**Table S6** Pairwise genetic differentiation (Cockerham and Weir’s ([1993](#_ENREF_1)) *β* statistic) between climate treatment sub-populations of *F. ovina* and *P. lanceolata* at BCCIL.

|  | Control | Drought | Heated | Watered | Heated/ Drought | Heated/ watered |
| --- | --- | --- | --- | --- | --- | --- |
| ***Festuca ovina*** | | | | | | |
| Control | — | 0.00594 | 0.00569 | 0.00702 | 0.00593 | 0.00556 |
| Drought | 0.00594 | — | 0.00701 | 0.00517 | 0.00727 | 0.00662 |
| Heated | 0.00569 | 0.00701 | — | 0.00312 | 0.00575 | 0.00433 |
| Watered | 0.00702 | 0.00517 | 0.00312 | — | 0.00656 | 0.00465 |
| Heated/ Drought | 0.00593 | 0.00727 | 0.00575 | 0.00656 | — | 0.00392 |
| Heated/ watered | 0.00556 | 0.00662 | 0.00433 | 0.00465 | 0.00392 | — |
| ***Plantago lanceolata*** | | | | | | |
| Control | — | 0.00805 | 0.00907 | 0.01591 | 0.01558 | 0.00745 |
| Drought | 0.00805 | — | 0.01225 | 0.01726 | 0.01715 | 0.00786 |
| Heated | 0.00907 | 0.01225 | — | 0.00718 | 0.00854 | 0.00943 |
| Watered | 0.01591 | 0.01726 | 0.00718 | — | 0.00328 | 0.01397 |
| Heated/ Drought | 0.01558 | 0.01715 | 0.00854 | 0.00328 | — | 0.01147 |
| Heated/ watered | 0.00745 | 0.00786 | 0.00943 | 0.01397 | 0.01147 | — |

**Text S7** structure analysis

*Methods*

Genetic composition of the population of each study species at BCCIL was assessed using the software structure v2.3.4 ([Pritchard *et al.*, 2000](#_ENREF_10)), which permits inference of the probable number of genetic clusters (*K*) present in the sampled population. Genetic structure was assessed using admixture models incorporating prior information on climate treatment sub-population membership ([Hubisz *et al.*, 2009](#_ENREF_6)). These models consider that individuals from the same sub-population may be more likely to share ancestry, and this prior information can assist in the clustering when genetic structure is weak. We ran 10 replicate structure analyses for each value of *K* from 1 to 10, using default settings for the Markov chain and other parameters. The most likely number of genetic clusters was assessed using the ∆*K* approach described by Evanno *et al*. ([2005](#_ENREF_3)), and implemented via the structure harvester platform ([Earl & von Holdt, 2012](#_ENREF_2)). Results from replicate structure analyses were combined using clumpp ([Jakobsson & Rosenberg, 2007](#_ENREF_7)) , using the Greedy algorithm (similarity coefficients were > 0.98).

*Results and conclusions*

Analysis using structure and the ∆*K* approach indicated *K* = 2 as the most likely number of genetic clusters for each study species (Figure S10c, d). However, log likelihood values for *K* = 1 and *K* = 2 were very similar for both datasets (Figure S10a, b; a structure of *K* = 1 cannot be tested using the Evanno ([2005](#_ENREF_3)) method). For *F. ovina*, the structure analysis provided evidence of a single genetic cluster that dominated the composition of all climate treatment sub-populations (Figure S11a). The second cluster was represented by an admixture signal within individuals. For *P. lanceolata*, the cluster membership of each of the climate treatment sub-populations was also dominated by a single cluster, with the second cluster represented by only a small proportion of individuals within several climate treatment sub-populations (Figure S11b). This latter genetic cluster was associated largely with individuals from experimental block C. These results suggest that the AFLP genotype data for each species are consistent with the presence of either one or two genetic clusters; genetic structure in these datasets is weak. In addition, these analyses do not provide evidence that genetic structure is associated with the long-term climate treatment manipulations imposed at BCCIL.

**Figure S8** Graphical output from structure harvester for the Evanno ([2005](#_ENREF_3)) analysis to infer the probable number of genetic clusters. (a), (b) Log likelihood plots for 10 replicate structure runs for each of 10 values of *K* (1–10). (c), (d) Plots of ∆*K* for 10 replicate structure runs for each of 10 values of *K* (1–10).

**Figure S9** Results of structure analysis for *K* = 2 clusters. (a), (c) *F. ovina*; (b), (d) *P. lanceolata*. Each plot shows inferred cluster membership for each individual as a vertical bar, based on 10 replicate runs of structure. Bars with mixed shading indicate inferred admixture between genetic clusters. (a) and (b) arrange the individuals by climate treatment sub-population. (c) and (d) arrange the individuals by experimental block.

**References**

Cockerham CC, Weir BS (1993) Estimation of gene flow from F-statistics. *Evolution,* **47**, 855–863.

Earl DA, Von Holdt BM (2012) STRUCTURE HARVESTER: a website and program for visualizing STRUCTURE output and implementing the Evanno method. *Conservation Genetics Resources,* **4**, 359–361.

Evanno G, Regnaut S, Goudet J (2005) Detecting the number of clusters of individuals using the software STRUCTURE: a simulation study. *Molecular Ecology,* **14**, 2611–2620.

Frankham R (1995) Effective population-size/adult-population size ratios in wildlife – a review. *Genetical Research,* **66**, 95–107.

Grime JP, Fridley JD, Askew AP, Thompson K, Hodgson JG, Bennett CR (2008) Long-term resistance to simulated climate change in an infertile grassland. *Proceedings of the National Academy of Sciences of the USA,* **105**, 10028–10032.

Hubisz MJ, Falush D, Stephens M, Pritchard JK (2009) Inferring weak population structure with the assistance of sample group information. *Molecular Ecology Resources,* **9**, 1322–1332.

Jakobsson M, Rosenberg NA (2007) CLUMPP: a cluster matching and permutation program for dealing with label switching and multimodality in analysis of population structure. *Bioinformatics,* **23**, 1801–1806.

Lynch M, Milligan BG (1994) Analysis of population genetic structure with RAPD markers. *Molecular Ecology,* **3**, 91–99.

Palstra FP, Ruzzante DE (2008) Genetic estimates of contemporary effective population size: what can they tell us about the importance of genetic stochasticity for wild population persistence? *Molecular Ecology,* **17**, 3428–3447.

Pritchard JK, Stephens M, Donnelly P (2000) Inference of population structure using multilocus genotype data. *Genetics,* **155**, 945–959.

Vekemans X (2002) AFLP-SURV version 1.0. Laboratoire de Génétique et Ecologie Végétale, Université Libre de Bruxelles, Belgium.

Whitlock R, Hipperson H, Mannarelli M, Butlin RK, Burke T (2008) An objective, rapid and reproducible method for scoring AFLP peak-height data that minimizes genotyping error. *Molecular Ecology Resources,* **8**, 725–735.

Zhivotovsky LA (1999) Estimating population structure in diploids with multilocus dominant DNA markers. *Molecular Ecology,* **8**, 907–913.
