# Supplementary material for: Rapid genetic divergence in response to 15 years of simulated climate change
Source: Glob Chang Biol. 2015 Aug 27;21(11):4165–76. doi: 10.1111/gcb.12966 (PMC4975715; doi:10.1111/gcb.12966)
Supplement: Supplementary file 3 — File S11. Readme file for R functions. [file GCB-21-4165-s003.docx]

***AFLP_R_functions_v1.1 Raj Whitlock, November 2014***

**Readme file for R functions supplied as supporting information**

*Data format*

The default format used for AFLP data is a tab delimited text file with population names in the first column, sample IDs/ names in the second column and locus names in the top row. The rest of the input file should be populated with the AFLP fragment data. Fragment presence should be denoted by a “1”, fragment absence by “0” and missing data by “9” (remember to omit the quotes in your file). Input data files can also be used in aflpdat format (Ehrich 2006), in this case you must specify format = “aflpdat” (see specific guidance below).

*Disclaimer*

In writing this set of R functions I have aimed to provide a resource that is useful to, and useable by those analysing binary dominant marker datasets, such as AFLP data. Many of these functions have been in use over a number of years, and have been improved during the course of their use. However, these R-functions are provided as-is, with no guarantee for their accuracy or suitability to carry out population genetic or other analyses. It is the users’ responsibility to check that the output given is consistent and sensible, and that the methods implemented are appropriate to their study system.

If you have comments and queries regarding any of the material set out here, then please contact Raj Whitlock ([r.whitlock@liverpool.ac.uk](mailto:r.whitlock@liverpool.ac.uk)).

*Quick start*

# First you must read in your data. Remember to set the working directory for your input data if you are not specifying the full path.

mydata <- read.delim (“mydata.txt”, header=T)

# Load the AFLP population genetics functions into R:

source("/Path/to/file/AFLP_R-functions_Mol_Ecol.R")

# Compute allele frequencies:

z1 <- zhiv (mydata)

# Calculate expected heterozygosities for each population:

h1 <- Hj (mydata)

# Calculate expected heterozygosities for each population at a common sample size of individuals, using a random resampling approach:

h2 <- Hj_samp (mydata, size = 15, nsamp = 100)

# Calculate genetic differentiation among populations, for each locus, following Cockerham and Weir (1993):

b1 <- betastat (mydata)

# Carry out a permutation test to determine whether the observed mean genetic differentiation across loci is statistically significant:

b2 <- betatest (mydata, nperm = 1000)

# Create a matrix describing pairwise genetic differentiation among populations:

b3 <- beta.dist.matrix (mydata)

# Calculate Wright’s *F*_ST_:

f1 <- FST (mydata)

# Write some data out (e.g. allele frequencies) to a tab-delimited text file (called “z1.txt”):

write.table (z1, file="z1.txt", sep="\t", quote=F, na=””, col.names=T, row.names=T)

**Function zhiv**

*Description*

Calculates null allele frequencies and their standard errors from AFLP or other dominant marker data, using the methods of Zhivotovsky (1999). Allele frequencies are computed for every locus in each population specified by the data, or for each locus globally (treating the dataset as a single population), depending on settings.

*Usage*

zhiv (x, prior = "non-uniform", prior.method = "within-pop", global = "F", format = "default")

*Arguments*

x An object of class data.frame containing the AFLP data, format as specified above.

prior Which prior should be used for the allele frequencies? Options are “non-uniform” or “uniform”. The uniform prior gives a uniform prior distribution on the null allele frequency *q*. The non-uniform prior uses prior information derived from either the distribution of fragment frequencies at the same locus in different populations, or from across different loci in a single population.

prior.method Where should prior information on allele frequencies be drawn from? If set to “among-pop”, then prior information on allele frequency for each locus is drawn from the distribution of fragment frequencies among populations at that locus. If set to “within-pop”, then prior information on allele frequency is drawn from the distribution of fragment frequencies among loci within each population.

global If TRUE, then the information on population membership is ignored in order to give a global estimate of allele frequencies. If FALSE, then population data are used and allele frequencies for each locus within each population will be returned.

format If set to “default”, then the default data format will be assumed. If set to “aflpdat”, then the aflpdat data format will be assumed.

*Details*

Allele frequencies are estimated following methods described in Zhivotovsky (1999). Missing data are ignored, and therefore sample sizes underpinning the allele frequency estimates may vary from locus to locus within populations, depending on the presence of missing data. The implementation used here assumes *F*_IS_ = 0; currently there is no facility to incorporate *F*_IS_ > 0.

*Value*

The method returns a matrix of null allele frequencies and standard errors with dimensions 2 * npop by nloc, where npop is the number of populations, and nloc is the number of loci. The first npop rows are populated by allele frequencies, and the following npop rows contain corresponding standard errors. Columns correspond to results for each locus.

*Example*

z1 <- zhiv (mydata)

*Author*

Dr Raj Whitlock [r.whitlock@liverpool.ac.uk](mailto:riwhitlock@liverpool.ac.uk)

**Function Hj**

*Description*

Calculate the expected heterozygosity for each population, along with the estimated standard error and variance for these statistics.

*Usage*

Hj (x, format = "default")

*Arguments*

x An object of class data.frame containing the AFLP data, format as specified above.

format If set to “default”, then the default data format will be assumed. If set to “aflpdat”, then the aflpdat data format will be assumed.

*Details*

This function calculates the mean expected heterozygosity for each population according to Lynch and Milligan (1994; Equations 4a, 5), assuming Hardy–Weinberg equilibrium. The estimate for each population is an average of estimates for each AFLP locus. This function calls zhiv with default settings in order to estimate allele frequencies (data format specifications supplied to Hj will be respected). If you wish to change the defaults for zhiv, then you must edit the zhiv function.

*Value*

Hj returns a matrix with as many rows as there are populations in the input file, and three columns. The first column displays the expected heterozygosity estimates, and the second and third columns show standard error and variance estimates of the heterozygosity statistics, respectively. Population names and column labels are incorporated as row names and column headers via the dimnames attribute of the matrix.

*Example*

h1 <- Hj (mydata)

*Author*

Dr Raj Whitlock [r.whitlock@liverpool.ac.uk](mailto:riwhitlock@liverpool.ac.uk)

**Function Hj_samp**

*Description*

This function implements a random re-sampling method to estimate expected heterozygosity for each population at a common sample size that is less than the minimum sample size in any of the populations.

*Usage*

Hj_samp (x, size, nsamp, format = "default")

*Arguments*

x An object of class data.frame containing the AFLP data, format as specified above.

size The number of individuals that will be sampled randomly from the total set of individuals genotyped in each population.

nsamp The number of replicate random samples to be taken.

format If set to “default”, then the default data format will be assumed. If set to “aflpdat”, then the aflpdat data format will be assumed.

*Details*

size must be lower than the minimum number of individuals genotyped in any of the populations present in the input file. Random samples of individuals from each population are taken without replacement (this process is repeated nsamp times). Heterozygosity and its standard error and variance are computed for each of the nsamp samples of size size, using function Hj, and the mean of these statistics is reported for each population.

*Value*

A data.frame with four columns containing population names, expected heterozygosity estimates and the estimated standard error and variance of the heterozygosity statistics.

*Example*

h2 <- Hj_samp (mydata, size = 15, nsamp = 100)

*Author*

Dr Raj Whitlock [r.whitlock@liverpool.ac.uk](mailto:riwhitlock@liverpool.ac.uk)

**Function betastat**

*Description*

Calculates genetic differentiation from dominant marker data, using Cockerham and Weir’s (1993) analogue of *F*_ST_ (*β*).

*Usage*

betastat (x, format = "default")

*Arguments*

x An object of class data.frame containing the AFLP data, format as specified above.

format If set to “default”, then the default data format will be assumed. If set to “aflpdat”, then the aflpdat data format will be assumed.

*Details*

This function will calculate Cockerham and Weir’s (1993) *β* statistic of population differentiation for each marker locus. Genetic differentiation is calculated over all populations specified in the input file. The sample size for the calculation of *β* (quantity *M*, in Cockerham & Weir 1993) is taken to be the mean of sample sizes for the populations specified in the input file. To create a matrix of pairwise differentiation, see function beta.dist.matrix. The function makes a call to zhiv with default settings, in order to calculate allele frequencies (data format specifications supplied to betastat will be respected).

*Value*

The function returns a data.frame with a single column containing population differentiation statistics, *β*. Locus names are given as the row.names of the data.frame.

*Example*

b1 <- betastat (mydata)

*Author*

Dr Raj Whitlock [r.whitlock@liverpool.ac.uk](mailto:riwhitlock@liverpool.ac.uk)

**Function betatest**

*Description*

This function carries out one- or two-sided permutation tests for summary statistics of genetic differentiation (mean or median across loci), using Cockerham and Weir’s (1993) *β* statistic of differentiation.

*Usage*

betatest (x, nperm = 1000 ,locus.summary = "mean", type = "two-tailed", format = "default")

*Arguments*

x An object of class data.frame containing the AFLP data, format as specified above.

nperm How many random permutations of individuals among populations should be made (integer)?

locus.summary Should population differentiation be summarized using the mean (locus.summary = “mean”), or the median (locus.summary = “median”)?

type Should the *p*-value returned by the function correspond to a one-tailed test (type = “one-tailed”) or a two-tailed test (type = “two-tailed”)?

format If set to “default”, then the default data format will be assumed. If set to “aflpdat”, then the aflpdat data format will be assumed.

*Details*

Random permutations of individuals among populations are used to create a null distribution for the population differentiation summary statistic. The observed value of genetic differentiation is included as a datum in the set of simulated values. The observed value is compared to the null distribution in order to derive the *p*-value. This function makes calls to betastat and zhiv, accepting default arguments for these functions (data format specifications supplied to betatest will be respected).

*Value*

The function returns a vector of length nperm, containing the null distribution for the summary statistic of population differentiation. The observed average population differentiation, the *p*-value, and quantiles of the permuted null distribution are all printed to the console.

*Example*

b2 <- betatest (mydata, nperm = 1000)

*Author*

Dr Raj Whitlock [r.whitlock@liverpool.ac.uk](mailto:riwhitlock@liverpool.ac.uk)

**Function beta.dist.matrix**

*Description*

Creates a pairwise genetic differentiation matrix among all population pairs, using Cockerham and Weir’s (1993) *β* statistic of differentiation.

*Usage*

beta.dist.matrix (mydata, format = "default")

*Arguments*

x An object of class data.frame containing the AFLP data, format as specified above.

format If set to “default”, then the default data format will be assumed. If set to “aflpdat”, then the aflpdat data format will be assumed.

*Details*

beta.dist.matrix is a wrapper for the functions given above that allows calculation of genetic differentiation among all pairs of populations present in an input file. This function makes calls to betastat and zhiv, accepting default arguments for these functions (data format specifications supplied to beta.dist.matrix will be respected).

*Value*

This function returns a symmetrical matrix containing genetic differentiation statistics (*β*), with dimensions (npop, npop), where npop is the number of populations. Population names are incorporated as the dimnames attributes of the returned matrix.

*Example*

b3 <- beta.dist.matrix (mydata)

*Author*

Dr Raj Whitlock [r.whitlock@liverpool.ac.uk](mailto:riwhitlock@liverpool.ac.uk)

**Function FST**

*Description*

Calculates an asymptotically unbiased estimate of Wright’s *F*_ST_ for dominant marker data, as described by Lynch and Milligan (1994).

*Usage*

FST (x, diversity.between = F, format = "default")

*Arguments*

x An object of class data.frame containing the AFLP data, format as specified above.

diversity.between If diversity.between = F, then *F*_ST_ is returned as a summary across loci. If diversity.between = T, then a matrix of between population heterozygosities is returned, along with a vector containing the mean within-population expected heterozygosity for each population.

format If set to “default”, then the default data format will be assumed. If set to “aflpdat”, then the aflpdat data format will be assumed.

*Details*

*F*_ST_ is calculated as specified in Lynch and Milligan (Equation 14a; 1994). Values of between-population gene diversity are given as heterozygosity in excess of that observed within populations (Equation 10a; Lynch & Milligan 1994). Values for within-population heterozygosity are given as unbiased estimates of expected heterozygosity (i.e. assuming Hardy–Weinberg equilibrium; Lynch & Milligan 1994, Equations 4a, 5). This function makes a call to zhiv in order to calculate allele frequencies, accepting default arguments for this function (data format specifications supplied to FST will be respected).

*Value*

If diversity.between = F, then the function returns a single numeric value for *F*_ST_. If diversity.between = T, then the function returns a list containing (1) a lower triangular matrix of between-population heterozygosities, and (2) a vector of within-population heterozygosities.

*Example*

f1 <- FST (mydata)

*Author*

Dr Raj Whitlock [r.whitlock@liverpool.ac.uk](mailto:riwhitlock@liverpool.ac.uk)

**References**

Cockerham CC, Weir BS (1993) Estimation of gene flow from F-statistics. *Evolution* **47**, 855–863.

Ehrich D (2006) AFLPdat: a collection of R functions for convenient handling of AFLP data. *Molecular Ecology Notes* **6**, 603–604.

Lynch M, Milligan BG (1994) Analysis of population genetic structure with RAPD markers. *Molecular Ecology* **3**, 91–99.

Zhivotovsky LA (1999) Estimating population structure in diploids with multilocus dominant DNA markers. *Molecular Ecology* **8**, 907–913.
